# Research on the application behavior of generative artificial intelligence learning of college students based on self-determination theory

**DOI:** 10.3389/fpsyg.2026.1805498

**Published:** 2026-04-01

**Authors:** Xu Fang, Junci Feng

**Affiliations:** College of Educational Sciences, Nantong University, Nantong, China

**Keywords:** deep processing, generative artificial intelligence, learning behavior, over-dependence, self-determination theory

## Abstract

**Introduction:**

In recent years, the application of generative artificial intelligence (GAI) in higher education has gained increasing prevalence, accompanied by a concerning rise in student “over-reliance”. Such excessive dependence can undermine critical thinking and hinder innovation development. Consequently, guiding college students toward a “deep processing” mode of GAI use has become a crucial issue in higher education.

**Methods:**

Grounded in self-determination theory, this study constructs a model to examine the factors influencing college students’ GAI usage behaviors. To test the proposed model and its hypothesized relationships, An empirical study has been conducted,including survey instrument distribution and path analysis.

**Results:**

The main findings emerge: First, among basic psychological needs, perceived competence plays a pivotal role in curbing over-reliance and promoting deep processing, exerting a stronger influence than perceived autonomy or relatedness. Second, all three basic psychological needs (perceived autonomy, perceived competence, and perceived relatedness) indirectly influence deep processing and over-reliance through intrinsic motivation.

**Discussion:**

The hypotheses proposed in the article has been verified. Based on these findings, practical suggestions are proposed across multiple levels to foster more reflective and self-determined GAI use, including constructing a need-supportive Learning Ecosystem, building a “guided” GAI tool, establishing a hierarchical support and constraint framework, fostering a shift from instrumental to alue rationality.

## Introduction

The rapid advancement and widespread adoption of technologies like DeepSeek and ChatGPT have demonstrated how Generative Artificial Intelligence (GAI) is fundamentally transforming higher education through its advanced content generation and interactive capabilities. Recognizing the transformative potential of Generative AI (GAI), nations worldwide have prioritized its integration into education, with policies promoting deep application to enhance learning (U.S. Department of Education, 2023; Cabinet Office, Government of Japan, 2019). However, this enthusiasm is accompanied by official caution against uncritical reliance. China’s recent “Opinions on Accelerating Educational Digitalization” explicitly aims to prevent issues like “addiction to dependence” (Central People’s Government, 2025a). Similarly, the OECD’s “Digital Education Outlook 2026” warns that students’ over-reliance on GAI can lead to “cognitive offloading” and undermine critical thinking (OECD, 2026). In practice, university students exhibit divergent usage patterns: some engage in deep, critical processing, while others fall into over-reliance (Zhang et al., 2024). Understanding the psychological drivers behind these behaviors is crucial to mitigate over-dependence and promote meaningful engagement.

Current research indicates a growing tendency among university students toward over-reliance on GAI for learning support (Bulawan et al., 2024). This dependency manifests in behaviors such as uncfritically submitting AI-generated outputs as final answers, using minimal or unrefined prompts, and prioritizing immediate AI assistance over independent problem-solving. Such superficial engagement risks diminishing students’ information retrieval skills and deep knowledge exploration (McNorgan et al., 2011; Biccheri et al., 2023), ultimately undermining critical thinking and creative problem-solving abilities, which are key competencies that higher education aims to cultivate.

These challenges emerge alongside heightened expectations for educational quality. China’s “Outline of the Educational Power Construction Plan (2024-2035)” (Central People’s Government, 2025b) explicitly calls for fostering higher-order thinking and lifelong learning capabilities in the AI era. This requires moving beyond basic tool utilization to more sophisticated engagement with GAI by designing effective prompts (Wang, 2021), critically evaluating AI outputs (Premkumar et al., 2024), and creatively synthesizing generated content (Fullan and Langworthy, 2014).

Given these imperatives, investigating the psychological and behavioral factors influencing students’ GAI usage patterns becomes both theoretically significant and practically urgent. Existing studies have identified several factors associated with students’ GAI usage behaviors. For example, inert thinking and avoidance learning motivation are positively linked to over-reliance (Ye et al., 2025; Zhai et al., 2024), while self-efficacy and performance expectancy also play important roles (Gazit, 2025; Jo, 2024). Furthermore, factors such as emotional support from the learning environment and users’ information-seeking characteristics have also been shown to influence students’ interactions with AI (Xian et al., 2025; Kim and Seo, 2025). Other research has explored factors influencing technology acceptance and behavioral intentions, including performance expectancy, social influence, and facilitating conditions (Lin and Jiang, 2025; Xu et al., 2025). Studies have also examined how individual differences such as personal innovativeness and habit shape technology adoption patterns (Wu et al., 2025; Lin and Jiang, 2025), and how contextual factors like task-technology fit and educational regulations influence learning outcomes with AI tools (Du and Lv, 2024; Bai, 2025). However, despite these valuable contributions, several important gaps remain. First, while existing research has identified a range of isolated predictors, systematic investigations are still scarce. Most studies treat technology use as a unidimensional construct, overlooking the fundamental differences in how students engage with GAI. Second, empirical research examining the motivational mechanisms underlying these divergent usage patterns remains underdeveloped. Few studies have empirically tested comprehensive models that explain why some students engage deeply while others become over-reliant. Third, there is a notable lack of research adopting an intrinsic motivation perspective to understand students’ GAI usage behaviors. The role of internally driven motivation in shaping how students interact with GAI has been overlooked. Self-determination theory (SDT), as one of the most established theories of intrinsic motivation, explains how basic psychological needs (perceived autonomy, sense of competence, and perceived relatedness) shape behavior through intrinsic motivation (Ryan and Deci, 2000a, 2000b), providing an integrated framework for understanding the divergence between deep processing and over-reliance in GAI learning contexts. Grounded in self-determination theory, this study develops an empirical model that identifies key determinants of deep processing versus over-reliance behaviors among university students by examining group-specific variations. Theoretically, this research systematically investigates the intrinsic motivational mechanisms underlying student engagement with AI technologies from the perspective of self-determination theory. By constructing a systematic framework that distinguishes between different behavioral patterns, it deepens the theoretical understanding of how psychological needs shape technology-mediated learning, addresses the lack of empirical research in this area, and provides an important theoretical lens and foundation for future studies. Practically, this research offers guidance for key stakeholders in higher education. It can help educators design tasks that support autonomy and deeper engagement with GAI, help administrators develop policies and training that promote responsible use, and help technology designers create tools that enhance students’ sense of control, confidence, and connection. Ultimately, the research aims to ensuring its use enhances rather than hinders the development of essential 21st-century skills.

## Theoretical basis

### Self-determination theory

Self-Determination Theory originally proposed by American psychologists Ryan and Deci (2000a, 2000b), establishes that human behavior is fundamentally motivated by three basic psychological needs: autonomy, competence, and relatedness (Gong, 2023). Within this framework, autonomy represents an individual’s need for volitional control over their actions, competence reflects the desire to effectively interact with and master one’s environment, and relatedness encompasses the need to establish meaningful social connections. Importantly, when these psychological needs are supported by external environments, they serve to both enhance intrinsic motivation and facilitate the internalization of extrinsic motivation.

The enhancement of intrinsic motivation occurs when individuals experience genuine autonomy, perceive opportunities for skill development, and establish social connections during activities, resulting in more spontaneous and engaged participation. Concurrently, the internalization process transforms externally regulated behaviors into self-determined actions as they become gradually aligned with personal values through continued need satisfaction. Within educational contexts, SDT has proven particularly valuable for understanding the complex interplay between learners’ motivational internalization processes, behavioral persistence, and affective experiences (Li et al., 2025).

Current scholarly applications of SDT to learning environments can be broadly categorized into three research streams. The first examines motivational mechanisms within technology-enhanced learning environments, with particular emphasis on platforms such as MOOCs, VR systems, and mobile learning applications. Empirical evidence from MOOC environments demonstrates that autonomy-supportive structures and competence-enhancing feedback mechanisms significantly influence both learning engagement and satisfaction outcomes (Liu et al., 2023). Similarly, research in VR instructional settings reveals how the dynamic interaction between perceived usability and self-determination needs generates distinct learning configurations, including technology-trust, value-guided, and self-determined learning modalities (Shi, 2025).

The second research stream investigates motivational enhancement through gamification strategies, where SDT principles inform instructional design decisions. David and Weinstein’s (2024) work illustrates how maintaining an optimal balance between challenge and skill development (competence support) while avoiding excessive competitive structures (autonomy undermining) can optimize learning motivation. Parallel investigations in augmented and virtual reality environments further demonstrate how exploratory learning designs can effectively nurture autonomous learning orientations (Erdoğmuş and Korkmaz, 2022).

The third research direction focuses on teacher support strategies informed by SDT principles. Guay’s (2022) findings reveal that instructors’ use of autonomy-supportive language, characterized by the provision of meaningful choices rather than directives, significantly enhances students’ motivation to engage with educational technologies. Extending this line of inquiry, Monacis et al. (2023) developed an Italian adaptation of the Learning Climate Questionnaire specifically designed to assess special education teachers’ implementation of need-supportive practices, thereby demonstrating SDT’s cross-cultural applicability in diverse educational settings.

This study’s application of SDT to analyze university students’ engagement with generative AI in learning contexts is theoretically justified by three key considerations. First, the theory’s comprehensive framework encompassing autonomy, competence, and relatedness provides a systematic approach for examining the motivational dynamics underlying technology adoption behaviors. Second, the psychological needs emphasized by SDT demonstrate particular congruence with the unique affordances of generative AI technologies, thereby offering valuable insights into the psychological mechanisms driving technology acceptance processes. Finally, SDT’s established explanatory power in digital learning environments positions it as an ideal theoretical foundation for informing the design of motivationally supportive educational AI applications.

### GAI usage behavior theory

Current research has identified two distinct behavioral patterns in college students’ utilization of GAI within learning contexts: deep processing-oriented usage and over-reliance-oriented usage (Hou et al., 2025).

Deep processing-oriented usage behavior characterizes students who engage with GAI in a critically reflective manner. These learners demonstrate active cognitive engagement by carefully formulating queries to elicit optimal outputs, critically evaluating AI-generated content, and employing GAI to refine initial drafts or solutions (Hou et al., 2025). Such reflective engagement aligns with established theories of metacognition, wherein individuals systematically assess their strategies, monitor progress, and adapt their approaches accordingly (Gencel and Saracaloğlu, 2018). Empirical evidence further supports that reflective thinking enhances cognitive presence and positively correlates with academic performance (Kim and Lim, 2019). When applied to GAI usage, this reflective behavior fosters rigorous self-regulation in problem-solving, thereby deepening cognitive engagement and improving learning outcomes.

Conversely, over-reliance-oriented usage behavior manifests as excessive dependence on GAI accompanied by diminished cognitive effort. Students exhibiting this behavior tend to submit instructor-assigned tasks or internet-sourced content directly to GAI without modification, uncritically adopting AI-generated outputs as their own solutions. This practice reflects deficiencies in independent critical thinking and information verification (Zhou and Li, 2023; Wang et al., 2025). From a knowledge acquisition perspective, such over-reliance promotes superficial learning by prioritizing rapid access to information over meaningful understanding. Consequently, it reduces students’ motivation for active knowledge exploration, thereby limiting both the depth and breadth of their learning (Liu and Zhang, 2024). This behavioral pattern not only impedes the development of effective technology-mediated learning strategies but also undermines the foundational cognitive skills necessary for creative thinking and long-term academic growth.

### Research hypotheses and construction of hypothetical model

**Figure 1:**
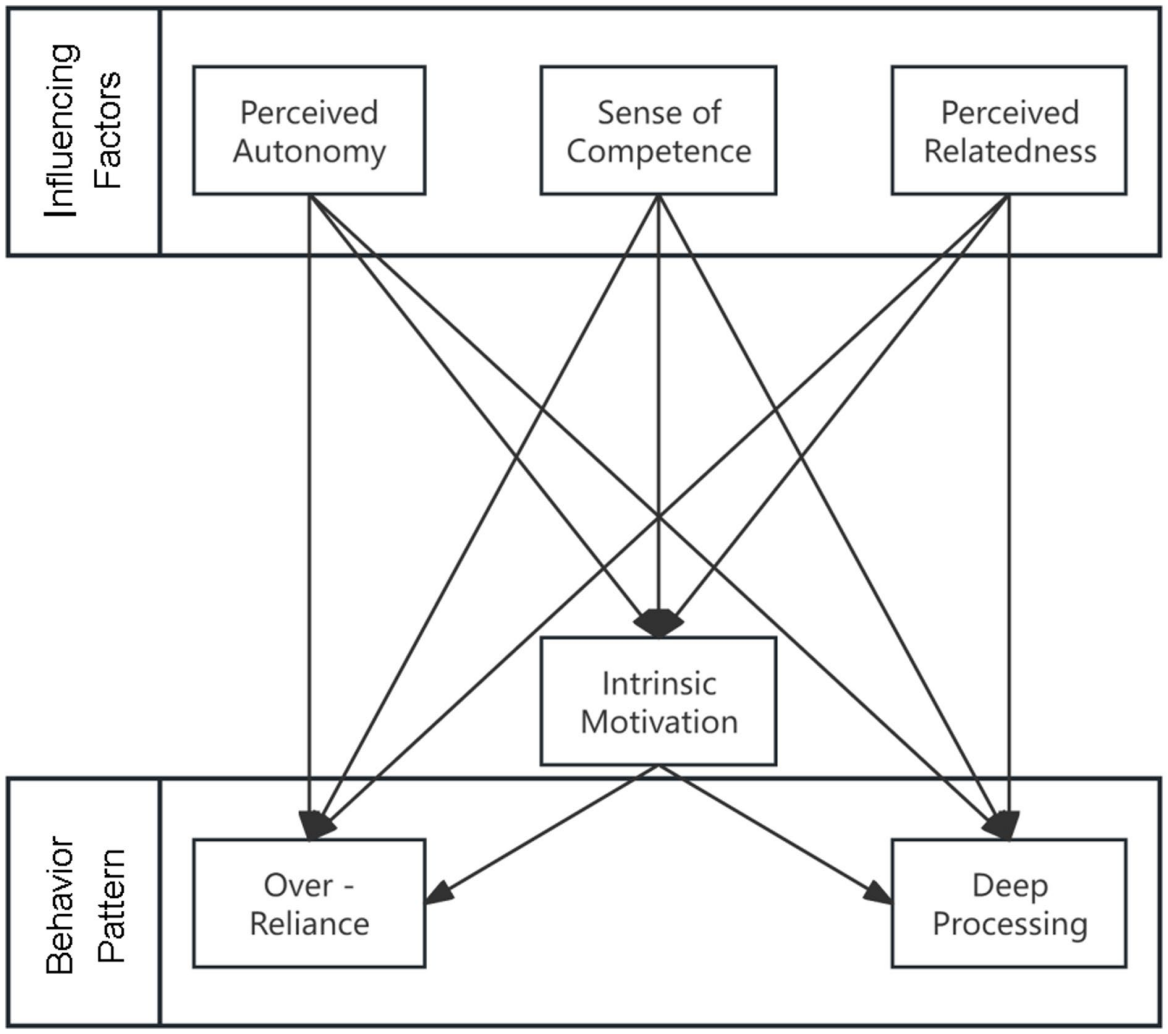
Model of influencing factors on college students’ GAI usage behavior. This figure presents a theoretical model illustrating how perceived autonomy, competence, and relatedness influence intrinsic motivation, which in turn shapes behavior patterns such as reliance, over-processing, or deep engagement with generative AI.

Drawing upon Self-Determination Theory and GAI usage behavior literature, this study develops a comprehensive conceptual model (Figure 1) that extends the SDT framework by distinguishing between deep processing and over-reliance as distinct behavioral outcomes and examining how autonomy, competence, and relatedness differentially predict these patterns through intrinsic motivation. In the context of college students’ GAI learning applications, their behavioral engagement is influenced by both extrinsic and intrinsic motivational factors, with intrinsic motivation playing a particularly crucial role in shaping meaningful learning experiences. Self-determination theory posits that the satisfaction of three basic psychological needs—autonomy, competence, and relatedness—enhances intrinsic motivation, which in turn guides individuals’ behavioral choices and engagement patterns. Specifically, when students experience a sense of volitional control in using GAI (perceived autonomy), feel confident in their ability to operate the technology effectively (sense of competence), and perceive supportive connections with peers and instructors during GAI use (perceived relatedness), their intrinsic motivation is strengthened, leading them to engage in deeper cognitive processing rather than falling into superficial over-reliance. Grounded in the three basic psychological needs as theoretical foundations, the model synthesizes GAI’s technological attributes with educational application contexts to elucidate the underlying mechanisms and pathways influencing students’ adoption of different GAI usage behaviors. Through systematic literature review and theoretical integration, this framework aims to provide a nuanced understanding of how intrinsic motivational factors interact with technological and contextual elements to shape either deep processing-oriented or over-reliance-oriented GAI usage patterns in higher education settings.

### The direct impact of perceived autonomy on behavioral patterns

Perceived autonomy, as a key dimension of basic psychological needs, directly shapes an individual’s behavioral regulation mode towards GAI. This concept reflects a state where users experience independent decision-making and control during GAI use, free from external coercion and based on their own needs and wishes. When students have a high level of perceived autonomy, their enhanced sense of control over the learning process can mitigate the risk of mechanical dependence, as they are more inclined to regard artificial intelligence as an auxiliary tool rather than a substitute. Empirical studies indicate that in the educational context of GAI, the satisfaction of autonomy needs correlates positively with its perceived usefulness (Chai et al., 2022). Furthermore, autonomy-supportive teaching environments have been shown to moderate behaviors linked to over-reliance, such as reducing premeditated cheating by fostering intrinsic honesty (Bureau et al., 2022). On the contrary, in low-autonomy situations, external pressure may trigger passive decision-making, potentially leading to over-reliance. This mechanism is rooted in the activation of cognitive resources by autonomy: research suggests that high autonomy can promote in-depth information processing because it stimulates individuals to invest energy in verifying GAI outputs rather than merely accepting them superficially (Luo et al., 2021). This fundamental capacity for self-directed engagement, positions perceived autonomy as a critical antecedent to deep, critical processing of information. Therefore, this study posits that perceived autonomy directly influences the two key behavioral patterns in GAI use.

*H1a*: Perceived autonomy has a significant negative impact on excessive reliance.

*H1b*: Perceived autonomy has a significant positive impact on deep processing.

### The direct impact of sense of competence on behavioral patterns

The sense of competence, corresponding to the need for ability, refers to an individual’s confidence in using GAI effectively and their perceived self-efficacy. This concept encompasses self-assessment in operational skills, result discrimination, and goal achievement, reflecting one’s overall confidence in completing tasks and overcoming challenges with GAI. Students with a high sense of competence, supported by solid knowledge schemas and confidence in their skills, can effectively evaluate the rationality of GAI outputs, conduct corrections, and perform cross-validation. Research indicates that a sense of competence in a given field reduces learning resistance and enhances motivation for subsequent independent exploration (Chik, 2014). In technology-enhanced learning contexts specifically, self-efficacy in using digital tools is a strong predictor of learning engagement (Wang and Huang, 2022; Hsiao, 2021). For instance, successful experiences in using technology to solve problems boost learners’ self-efficacy, thereby strengthening their motivation to explore the technology independently (Jacobs and Castek, 2018). Studies on generative AI in education show that perceived competence strengthens intrinsic motivation and the willingness to integrate information (Jo, 2024). Broader psychological research further supports that self-efficacy facilitates critical reflection and that a disposition toward critical thinking is associated with higher self-efficacy (Sekerdej and Szwed, 2021; Mangion et al., 2024). Conversely, students with a low sense of competence may doubt their own abilities, leading them to avoid cognitive challenges. This can result in a tendency to rely directly on GAI to complete assignments, forming a pattern of excessive reliance. Empirical studies in language learning contexts have found that students with insufficient confidence may bypass deep processing and directly depend on GAI for tasks like writing (Du and Alm, 2024; Munoz et al., 2025). Therefore, a student’s perceived competence is posited to directly influence their behavioral engagement with GAI.

*H2a*: Sense of competence has a significant negative impact on excessive reliance.

*H2b*: Sense of competence has a significant positive impact on deep processing.

### The direct impact of perceived relatedness on behavioral patterns

Perceived relatedness corresponds to the need for connection, referring to an individual’s perception of support and linkage within their social environment during GAI use. It manifests as establishing and maintaining supportive relationships with peers and instructors, fostering a sense of belonging and reciprocal support that can continuously promote engagement. In collaborative learning environments, students with a high level of perceived relatedness tend to view GAI as a “learning partner” and continuously optimize their usage through interactions with teachers and peers (Wu, 2018). The social support provided by these interactions can effectively alleviate students’ anxiety about technology reliance, prompting them to proactively examine potential information biases in GAI outputs, thereby reducing the probability of excessive reliance behaviors (Brewer and Klein, 2006; Li et al., 2024; Zhou et al., 2025). Furthermore, when students’ relatedness needs are well satisfied, it stimulates knowledge-sharing behaviors. For example, students are more inclined to discuss GAI-generated content with peers or deepen their understanding under teacher guidance. Such social processing naturally fosters deep learning. Conversely, if students are in an isolated environment lacking opportunities for social connection and verification, they may become overly reliant on the immediate feedback from GAI. This lack of social scaffolding can strengthen a one-way dependence on the technology, as the absence of collaborative norms diminishes a key counterbalance to technical system dominance. Therefore, the degree of perceived relatedness within the learning context is hypothesized to directly shape how students engage with GAI.

*H3a*: Perceived relatedness has a significant negative impact on excessive reliance.

*H3b:* Perceived relatedness has a significant positive impact on deep processing.

### The impact of perceived autonomy on behavioral patterns through intrinsic motivation

According to self-determination theory, satisfying the basic psychological need for autonomy enhances an individual’s intrinsic motivation for learning. In the context of GAI, when users perceive they have independent decision-making and control over the process—such as in choosing the time, content, and goals of use—this experience of “perceived autonomy” fulfills their need for self-determination, thereby strengthening their internal drive to engage with the technology (Ryan and Deci, 2000a, 2000b). Educational research consistently supports that autonomy support is a pivotal factor in fostering students’ intrinsic motivation across various learning environments, including those enhanced by technology (Descals-Tomás et al., 2021; Chiu et al., 2023). Furthermore, studies exploring AI applications in higher education have found a significant positive correlation between users’ perceived autonomy and their willingness to use such technologies (Niu et al., 2024; Yang et al., 2022). This enhanced intrinsic motivation, in turn, is associated with subsequent behavioral patterns. Self-determination theory posits intrinsic motivation as the natural drive for self-determined learning behaviors (Ryan and Deci, 2000a, 2000b), which translates into a reduced tendency to seek quick, unreflective answers from GAI, thereby mitigating excessive reliance (An et al., 2024). Concurrently, intrinsically motivated learners exhibit greater cognitive engagement and are more likely to employ deep processing strategies, investing effort into understanding and critically evaluating information (Sins et al., 2008). Enjoyment as a core affective component of intrinsic motivation has been shown to mediate the relationship between positive technology experiences and continued use intention (Li, 2025). The pivotal role of intrinsic motivation in mediating the link between technology acceptance and deeper learning engagement has been empirically validated (Urban and Urban, 2023; Wang and Pan, 2025). Wang and Liu (2025), in their recent study on AI-supported creative learning, also highlighted that intrinsic motivation drives deeper engagement and perceived learning outcomes. Therefore, it is theorized that perceived autonomy influences how students use GAI not only directly but also indirectly by first cultivating a stronger intrinsic motivation, which then channels their behavior towards either deeper processing or away from over-reliance.

*H4a*: Perceived autonomy has a significant negative impact on excessive reliance through the mediation of intrinsic motivation.

*H4b*: Perceived autonomy has a significant positive impact on deep processing through the mediation of intrinsic motivation.

### The impact of sense of competence on behavioral patterns through intrinsic motivation

The pivotal role of perceived competence is further underscored by research showing that self-efficacy significantly predicts both students’ AI readiness and academic performance in AI-mediated learning contexts (Wang and Li, 2024). When students perceive themselves as competent in operating the technology, discriminating its outputs, and managing associated tasks, this positive self-assessment fulfills their need for efficacy. Research indicates that such competence satisfaction reduces resistance to learning and actively enhances an individual’s motivation for subsequent independent exploration (Chik, 2014). This is particularly salient in technology-enhanced contexts, where self-efficacy in using digital tools has been demonstrated to be a strong predictor of learning motivation and engagement (Wang and Huang, 2022; Hsiao, 2021). For example, successful experiences in using technology to solve problems reinforce learners‘self-efficacy, thereby strengthening their drive to engage with and explore the technology independently (Jacobs and Castek, 2018). This confidence and the resultant intrinsic motivation are pivotal in shaping specific behavioral engagements with GAI. Intrinsic motivation, as outlined by self-determination theory, serves as a natural drive for self-determined behavior (Ryan and Deci, 2000a, 2000b). In the GAI learning context, this internal drive reduces the tendency to seek quick, unreflective answers, thereby exerting a negative influence on excessive reliance (An et al., 2024). Concurrently, learners with high intrinsic motivation exhibit more cognitive engagement and are more likely to employ deep processing strategies, investing effort in understanding, integrating, and critically evaluating information (Sins et al., 2008). The role of intrinsic motivation as a core mediator in the pathway from technology acceptance to meaningful learning behavior has received empirical support (Urban and Urban, 2023; Wang and Pan, 2025). Consequently, it is proposed that a sense of competence influences behavioral patterns not only directly but also via the enhancement of intrinsic motivation, which subsequently guides behavior towards deeper processing and away from over-reliance.

*H5a*: Sense of competence has a significant negative impact on excessive reliance through the mediation of intrinsic motivation.

*H5b*: Sense of competence has a significant positive impact on deep processing through the mediation of intrinsic motivation.

### The impact of perceived relatedness on behavioral patterns through intrinsic motivation

Perceived relatedness reflects the sense of belonging and reciprocal support derived from establishing and maintaining quality relationships within the learning environment, a factor consistently emphasized for its role in sustaining engagement. Studies have shown that positive interactions with teachers and peers are positively correlated with the intrinsic motivation for self-regulated learning (Qa et al., 2023; Ebadi and Amini, 2022). When students feel connected and supported, their basic psychological need for relatedness is satisfied, which in turn enhances their internal drive to explore and learn with GAI. This intrinsic motivation, fostered by relatedness satisfaction, subsequently acts as a critical regulator of behavior. According to the core propositions of self-determination theory, intrinsic motivation serves as the natural drive for self-determined behaviors (Ryan and Deci, 2000a, 2000b). Within GAI learning, engagement driven by genuine interest reduces the tendency to opt for quick, unreflective answers, thereby negatively influencing the development of excessive reliance (An et al., 2024). Simultaneously, intrinsically motivated learners are characterized by greater cognitive engagement and a higher propensity to employ deep processing strategies, directing effort toward understanding, integrating, and critically evaluating information rather than accepting it superficially (Sins et al., 2008; Chen and Barger, 2016). Empirical research substantiates the pivotal mediating role of intrinsic motivation in translating positive perceptions and acceptance of technology into deeper, more effortful learning engagement (Urban and Urban, 2023; Wang and Pan, 2025). Thus, it is hypothesized that perceived relatedness exerts its influence on how students interact with GAI along an indirect pathway: by first strengthening intrinsic motivation, which then channels behavioral outcomes toward deeper processing and away from over-reliance.

*H6a*: Perceived relatedness has a significant negative impact on excessive reliance through the mediation of intrinsic motivation.

*H6b*: Perceived relatedness has a significant positive impact on deep processing through the mediation of intrinsic motivation.

## Empirical analysis

### Survey instrument design

This study collects data by administering a survey instrument to college students. To ensure the quality of the survey instrument, a 5-point Likert scale is adopted in the design of the measurement scale. The items in the survey instrument are all derived from classic theories. Except for basic information, they are divided into several variables including “intrinsic motivation,” “sense of competence”, “perceived autonomy”, “perceived relatedness”, “over-reliance” and “deep processing”, which correspond to the hypothetical model. After initially setting the items, three experts in the field of educational technology were invited to revise them, and 50 college students were selected for a pre-survey. Based on the results, unreasonable items were deleted and adjusted to form the final official version of the survey instrument. The final items of the survey instrument and their reference sources are shown in Table 1 below.

**Table 1:** Survey instrument items and reference sources.

Variable	Items	Reference sources
Intrinsic Motivation (IM)	IM1: I use GAI because of interest	Lee and Davis (2024) and Quibuen et al. (2025)
	IM2: I use GAI because I like it	
Sense of Competence (SC)	SC1: I feel capable of using GAI well	Hidayat et al. (2025), Quibuen et al. (2025), and Ivanov et al. (2024)
	SC2: Using GAI in learning gives me a sense of accomplishment	
	SC3: I feel capable of distinguishing the results generated by GAI	
	SC4: I feel capable of using GAI correctly to achieve the goal of improving learning	
	SC5: I feel capable of avoiding risks brought by using GAI, including ethical and political risks	
Perceived Autonomy (PA)	PA1: In the process of using GAI, I can decide the usage time independently	Silva et al. (2024) and Tanantong and Wongras (2024)
	PA2: In the process of using GAI, I can decide the content to use independently	
	PA3: In the process of using GAI, I can independently choose which GAI product to use	
	PA4: In the process of using GAI, I can decide the usage progress independently	
	PA5: In the process of using GAI, I can set usage goals independently	
Perceived Relatedness (PR)	PR1: I maintain a good relationship with other students who use GAI together	Kang et al. (2024) and Xie et al. (2022)
	PR2: I maintain a good relationship with teachers who support my use of GAI	
Over-Reliance (ER)	ER1: I copy task descriptions or questions to GAI to seek its help	Loján et al. (2024) and Zhang et al. (2023)
	ER2: I will directly copy GAI’s answers as the answers to my questions	
	ER3: When I encounter problems, I mostly turn to GAI for help	
	ER4: I adopt GAI’s inputs without modification	
Deep Processing (DP)	DP1: I design my own prompts so that GAI can provide me with answers	Hu et al. (2025), Gajos and Mamykina (2022), and Hou et al. (2025)
	DP2: I usually carefully distinguish the correctness of GAI-generated answers and then use them selectively	
	DP3: When GAI provides me with a more appropriate answer, I will revise my original answer	
	DP4: I use GAI to improve my original answers	

### Data collection

This study collected data from college students through the Wenjuanxing platform by distributing the survey instrument via multi-level distribution channels to cover samples from universities of different levels and disciplines across the country. At the initial stage, a total of 821 completed instruments were recovered. Subsequently, a three-stage data cleaning process of “completeness screening - outlier detection and handling - duplicate response checking” was implemented to improve data quality and lay a reliable foundation for subsequent analysis. Finally, 750 valid responses to the survey instrument were obtained, with an effective recovery rate of 91.4%.

## Reliability and validity test

### Reliability test

SPSS 25 was used to conduct reliability analysis on the data collected from the survey instrument. First, an overall reliability analysis was performed, and the reliability coefficient value was 0.893, which is >0.8. According to Tavakol and Dennick (2011) research, when the *α* coefficient is >0.80, it indicates that the internal consistency is very good, thus showing that the reliability quality of the research data is high. Secondly, reliability analysis was conducted for each variable, and the results are summarized in Table 2. It can be seen from the table that the Cronbach’s α coefficients of all variables are >0.6, and most of them are >0.8. According to Düz Özer (2017) research, a Cronbach’s α coefficient ranging from 0.6 to 0.8 indicates moderate reliability, which shows that the reliability of each variable meets the requirements. Regarding the “α coefficient if item is deleted”, the reliability coefficient does not increase significantly after any item in the survey instrument is deleted, so it indicates that no item in the survey instrument should be deleted.

**Table 2:** Results of survey instrument reliability analysis.

Variable	Items	Corrected item-total correlation (CITC)	Alpha coefficient if the item is deleted	Cronbach’s alpha coefficient of each variable	Cronbach’s alpha coefficient of the entire survey instrument
Intrinsic Motivation (IM)	IM1	0.64	0.884	0.726	0.893
	IM2	0.647	0.884		
Sense of Competence (SC)	SC1	0.685	0.883	0.849	
	SC2	0.658	0.884		
	SC3	0.653	0.884		
	SC4	0.705	0.882		
	SC5	0.648	0.884		
Perceived Autonomy (PA)	PA1	0.686	0.883	0.852	
	PA2	0.677	0.883		
	PA3	0.668	0.884		
	PA4	0.663	0.884		
	PA5	0.675	0.883		
Perceived Relatedness (PR)	PR1	0.67	0.883	0.683	
	PR2	0.66	0.884		
Over-Reliance (ER)	ER1	−0.234	0.906	0.821	
	ER2	−0.316	0.908		
	ER3	−0.3	0.907		
	ER4	−0.391	0.909		
Deep Processing (DP)	DP1	0.7	0.883	0.832	
	DP2	0.666	0.884		
	DP3	0.663	0.884		
	DP4	0.701	0.883		
	DP5	0.669	0.884		

### Validity test

This study examined the validity of the instrument through confirmatory factor analysis, and the results showed that the overall construct validity was excellent. First, the KMO and Bartlett tests were conducted. As can be seen from Table 3, the KMO value reached 0.972, which is much higher than the threshold of 0.8, indicating that the data are very suitable for information extraction; the result of Bartlett’s test of sphericity was significant (*p <* 0.001), which fully supports that there is a strong correlation between variables, meeting the prerequisite for factor analysis (Field, 2013).

**Table 3:** KMO and Bartlett’s test.

KMO		0.972
Bartlett’s test of sphericity	Approx. Chi-Square	9529.308
	df	253
	*p*	0.000

This study subsequently adopted maximum likelihood estimation to conduct a confirmatory factor analysis on the model with 6 latent variables and 22 measurement items. The effective sample size was 750, meeting the basic requirement of exceeding 10 times the number of items. Analysis results indicated good overall model fit, although the reliability and validity of some dimensions require further examination. The model fit indices are presented in Table 4 and align well with the theoretical framework. The chi-square to degrees of freedom ratio (χ^2^/df) was 1.968, below the strict threshold of 3, suggesting a reasonable model structure. The goodness-of-fit index (GFI) was 0.956, indicating strong data explanation capability. The root mean square error of approximation (RMSEA) was 0.036 (90% CI: [0.031, 0.041]), significantly lower than the 0.08 cutoff and approaching the excellent standard of 0.04, indicating minimal fitting errors. The standardized root mean square residual (SRMR) was 0.037, within the ideal range. The comparative fit index (CFI) reached 0.979, the normed fit index (NFI) was 0.958, and the non-normed fit index (NNFI) was 0.975, all meeting criteria for an excellent model, confirming its superiority over the baseline model and parsimony. Although the chi-square test was significant, this should not be the primary criterion as large samples inflate the chi-square value. Considering robust indices like GFI, RMSEA, and CFI comprehensively, the model demonstrates excellent fit with well-controlled residuals and is generally acceptable.

**Table 4:** Model fit indicators.

Common indicators	*χ* ^2^	df		*χ*^2^/df	GFI	RMSEA	RMR	CFI	NFI	NNFI	TLI
Judgment Criteria	–	–	>0.05	<3	>0.9	<0.10	<0.05	>0.9	>0.9	>0.9	>0.9
Value	381.876	194	0.000	1.968	0.956	0.036	0.037	0.979	0.958	0.975	0.975

Next, convergent validity of the survey instrument was analyzed using confirmatory factor analysis (CFA). As presented in Table 5, all measurement items had significant standardized factor loadings (*p <* 0.001), ranging from 0.681 to 0.778, exceeding the benchmark of 0.60 (Hair et al., 2019). This indicates that each latent variable effectively explains its corresponding items. Specifically, the item “I adopt GAI’s input without modification” (Factor E) had the highest loading (0.778), while “I maintain a good relationship with my classmates” (Factor D) had the lowest, though still sufficient, loading of 0.722. Furthermore, all items’ squared multiple correlations (SMC) exceeded 0.40, ranging from 0.464 to 0.606, indicating that the latent variables explain over 40% of the variance in their items, confirming good convergent validity for the scale.

**Table 5:** Factor loading coefficient table.

Factor (latent variable)	Measurement item (observed variable)	*p*	Standardized load coefficient (std. estimate)	SMC
Intrinsic Motivation (IM)	IM1	–	0.753	0.568
	IM2	0.000	0.757	0.573
Sense of Competence (SC)	SC1	–	0.742	0.551
	SC2	0.000	0.706	0.499
	SC3	0.000	0.717	0.514
	SC4	0.000	0.758	0.574
	SC5	0.000	0.716	0.512
Perceived Autonomy (PA)	PA1	–	0.752	0.565
	PA2	0.000	0.727	0.529
	PA3	0.000	0.721	0.520
	PA4	0.000	0.725	0.526
	PA5	0.000	0.731	0.535
Perceived Relatedness (PR)	PR1	–	0.722	0.522
	PR2	0.000	0.718	0.515
Over-Reliance (ER)	ER1	–	0.681	0.464
	ER2	0.000	0.731	0.535
	ER3	0.000	0.731	0.534
	ER4	0.000	0.778	0.606
Deep Processing(DP)	DP1	–	0.754	0.568
	DP2	0.000	0.738	0.545
	DP3	0.000	0.722	0.522
	DP4	0.000	0.758	0.575

The horizontal bar ‘-’ indicates that this item is a reference item.

As shown in Table 6, the Average Variance Extracted (AVE) values for the latent variables range from 0.518 to 0.570, all exceeding the 0.50 threshold, supporting the basic convergent validity of each factor. Regarding Composite Reliability (CR), Factors B (0.849), C (0.852), E (0.821), and Factor 6 (0.832) all surpass the ideal level of 0.70. Although Factors A (CR = 0.726) and D (CR = 0.683) are slightly below 0.70, they remain within the acceptable range of ≥0.60, and both exhibit adequate AVE values (0.570 and 0.518, respectively). It is noteworthy that Factor D, being a two-item construct, typically yields lower CR values. Nevertheless, the obtained result indicates acceptable internal consistency (Kim et al., 2013).

**Table 6:** Results of model AVE and CR indicators.

Factor	Average variance extracted (AVE)	Composite reliability (CR)
A (Internal Motivation)	0.570	0.726
B (Sense of Competence)	0.530	0.849
C (Perceived Autonomy)	0.535	0.852
D (Perceived Relatedness)	0.518	0.683
E (Excessive Reliance)	0.534	0.821
F (Deep Processing)	0.553	0.832

Next, discriminant validity of the survey instrument was analyzed using the Fornell-Larcker criterion, with results presented in Table 7. The analysis confirms the discriminant validity of the instrument. First, basic validity standards are met: The square roots of AVE for all latent variables range from 0.720 to 0.755, significantly exceeding both the strict standard of 0.7 and the basic threshold of 0.5 (Fornell and Larcker, 1981), demonstrating excellent internal aggregation for each construct. For example, the square root of AVE for Factor A (Internal Motivation) is 0.755, higher than its maximum correlation coefficient of 0.712 (with Factor B). Similarly, for Factor E (Excessive Reliance), its square root of AVE (0.731) is markedly higher than the absolute values of all its correlation coefficients (the maximum absolute value being only 0.488), indicating particularly strong discriminant validity. Second, the observed correlation pattern aligns with theoretical logic: Factor E (Excessive Reliance) exhibits negative correlations with positive ability constructs, such as Sense of Competence B (r = −0.356) and Deep Processing F (r = −0.421). This pattern is consistent with the theoretical expectation that reliant behavior diminishes active cognitive participation, strongly supporting the core hypothesis that excessive reliance inhibits learning ability development and highlighting the scale’s criterion-related validity. Third, factor synergy effects reveal meaningful theoretical connections: Moderate correlations among some factors verify theoretical propositions, such as the link between need satisfaction and intrinsic motivation posited by self-determination theory. Finally, construct independence is established: Comparison of the square roots of AVE with the inter-factor correlation coefficients confirms that all latent variables satisfy the discriminant validity criterion (The square root of AVE > maximum correlation coefficient). This indicates measurement dimensionality independence and the absence of serious multicollinearity issues. Consequently, discriminant validity is established per the Fornell-Larcker criterion. The observed correlation patterns provide empirical support for the theoretical hypotheses, laying a reliable foundation for subsequent model analysis.

**Table 7:** Pearson correlations and square roots of AVE.

Factor	A	B	C	D	E	F
A (Internal Motivation)	0.755					
B (Sense of Competence)	0.712	0.728				
C (Perceived Autonomy)	0.683	0.612	0.731			
D (Perceived Relatedness)	0.657	0.534	0.534	0.720		
E (Excessive Reliance)	−0.411	−0.488	−0.446	−0.442	0.731	
F (Deep Processing)	0.659	0.604	0.582	0.539	0.445	0.743

The diagonal numbers represent the square root of AVE.

### Descriptive statistics

This study collected 750 valid responses to the survey instrument, with detailed demographic characteristics presented in Tables 8, 9. Regarding gender distribution, female respondents comprised 56.93% versus 43.07% male, consistent with the typically higher female participation in educational surveys. Undergraduates constituted the majority (90.40%), with sophomores representing the largest subgroup (25.60%). Postgraduate and doctoral students accounted for 6.80 and 2.80%, respectively. The sample covered all 12 disciplinary categories (including “Other”), with STEM fields and education prominently represented: science (15.60%), engineering (13.33%), and education (11.33%). Literature and medicine formed a secondary tier, while niche disciplines like philosophy and art were also included, reflecting comprehensive disciplinary diversity. Non-“Double First-Class” universities comprised the largest institutional category (63.47%), aligning with China’s higher education structure. “Double First-Class” institutions were fully represented, including world-class universities (16.27%) and those with first-class disciplines only (13.47%). Provincial universities (36.40%) and those directly under central ministries (30.13%) constituted the primary institutional types, followed by central-local jointly administered institutions (21.87%). Geographically, universities spanned eastern coastal (e.g., Jiangsu, Guangdong), central (e.g., Hubei, Hunan), western (e.g., Sichuan, Shaanxi), and northeastern regions (e.g., Liaoning, Jilin), demonstrating significant regional diversity. This demographically balanced sample provides a robust foundation for researching GAI’s educational applications, with a focus on undergraduates while encompassing diverse disciplines, institution tiers, and regions.

**Table 8:** Results of frequency analysis.

Name	Option	Frequency	Percentage (%)	Cumulative percentage (%)
Gender	Male	323	43.07	43.07
	Female	427	56.93	100.00
Grade	Freshman	160	21.33	21.33
	Sophomore	192	25.60	46.93
	Junior	181	24.13	71.07
	Senior	145	19.33	90.40
	Master’s Student	51	6.80	97.20
	Doctoral Student	21	2.80	100.00
Discipline	Philosophy	45	6.00	6.00
	Economics	44	5.87	11.87
	Law	39	5.20	17.07
	Education	85	11.33	28.40
	Literature	57	7.60	36.00
	History	39	5.20	41.20
	Science	117	15.60	56.80
	Engineering	100	13.33	70.13
	Agriculture	38	5.07	75.20
	Medicine	59	7.87	83.07
	Management	49	6.53	89.60
	Arts	38	5.07	94.67
	Others	40	5.33	100.00

**Table 9:** Summary table of response rate and popularization rate.

University level	Response		Popularization rate (*n* = 750)
	*n*	Response rate	
World-class University Construction Institutions, World-class Discipline Construction Institutions	122	8.96%	16.27%
Only World-class Discipline Construction Institutions	101	7.42%	13.47%
Non-world-class University, Non-world-class Discipline Construction Institutions	476	34.95%	63.47%
Institutions Directly Affiliated to National Ministries and Commissions	226	16.59%	30.13%
Provincial Local Institutions	273	20.04%	36.40%
Institutions Co-constructed by National Ministries and Local Governments	164	12.04%	21.87%

*χ^2^ =* 418.449, *p* = 0.000 in the goodness-of-fit test.

### Path analysis

This study conducted path analysis using SmartPLS 4 software, tested the model hypotheses through path analysis, and delved into the complex mechanism of action between variables. The path analysis results (Table 10) indicate that 11 out of the 12 hypothesized paths reached the statistical significance level (*p <* 0.05).

**Table 10:** Path analysis results.

Path	Original sample (*β*)	sample mean	Standard deviation	T statistics	*p*
Perceived Autonomy → Excessive Reliance	−0.076	−0.079	0.051	1.487	0.137
Perceived Autonomy → Deep Processing	0.272	0.273	0.046	5.904	0.000
Sense of Competence → Excessive Reliance	−0.273	−0.273	0.055	4.945	0.000
Sense of Competence → Deep Processing	0.382	0.382	0.046	8.365	0.000
Perceived Relatedness → Excessive Reliance	−0.144	−0.143	0.042	3.405	0.001
Perceived Relatedness → Deep Processing	0.223	0.222	0.039	5.665	0.000
Perceived Autonomy → Intrinsic Motivation → Excessive Reliance	−0.038	−0.039	0.016	2.375	0.018
Perceived Autonomy → Intrinsic Motivation → Deep Processing	0.053	0.053	0.021	2.524	0.012
Sense of Competence → Intrinsic Motivation → Excessive Reliance	−0.068	−0.068	0.025	2.72	0.007
Sense of Competence → Intrinsic Motivation → Deep Processing	0.094	0.094	0.031	3.032	0.002
Perceived Relatedness → Intrinsic Motivation → Excessive Reliance	−0.041	−0.041	0.017	2.412	0.016
Perceived Relatedness → Intrinsic Motivation → Deep Processing	0.057	0.057	0.023	2.478	0.013

## Discussion

### The direct effect of perceived autonomy on behavioral patterns (H1a and H1b)

The analysis reveals a direct effect of perceived autonomy on deep processing, with a significant positive path coefficient (*β* = 0.272), confirming the hypothesis (H1b). This suggests that users with higher perceived autonomy are more likely to activate metacognitive abilities and critically evaluate GAI outputs, positioning themselves as “evaluators” rather than passive recipients, which is associated with deeper cognitive engagement (Tankelevitch et al., 2024; Guidotti et al., 2018). Crucially, the degree to which perceived autonomy is internalized and integrated with personal values determines its optimal functioning in promoting meaningful engagement (Gao, 2016). However, the direct impact of perceived autonomy on excessive reliance was not significant (*β* = −0.076, *p* = 0.137), leading to the rejection of the direct effect hypothesis (H1a). Further analysis suggests that college students with high perceived autonomy may not form a homogeneous group. One subgroup appears to engage in deep processing by exercising their autonomy for critical use and verification, while another might leverage the same autonomy to optimize for efficiency and instrumental outcomes. These opposing behavioral tendencies within the high-autonomy population could potentially cancel each other out at the aggregate level, resulting in a non-significant overall direct relationship with overreliance (Ryan and Deci, 2000a, 2000b). Perceived autonomy can have dual effects, as its influence on behavior depends on how it is experienced and whether it is accompanied by other psychological needs (Haerens et al., 2015). This observation aligns with findings in technology-mediated learning, where autonomy can be channeled toward either deep mastery-oriented engagement or superficial efficiency-oriented use, depending on individual goals and contextual factors (Zhang and Chen, 2023; Peng, 2023). This finding suggests that educators can foster students’ critical engagement with GAI by creating autonomy-supportive learning environments and providing guidance on critical use (Guay, 2022).

### The direct effect of sense of competence on behavioral patterns (H2a and H2b)

The results strongly support the direct effects of a sense of competence on both behavioral patterns. It significantly promotes deep processing (*β* = 0.382) and inhibits excessive reliance (*β* = −0.273), verifying both direct effect hypotheses (H2b and H2a). This indicates that college student users with a higher sense of competence are more willing to actively explore the usage of GAI and engage in activities such as revising its outputs, rather than merely relying on provided answers (Xu and Ouyang, 2022). The findings align with the understanding that self-efficacy is a critical psychological resource. It is positively associated with the adoption of deeper learning strategies, yet it may also engage in a complex relationship with technological dependence, as high confidence could theoretically facilitate both mastery and efficiency-seeking behaviors (Hu and Yeo, 2020; Mielke, 2021; Zhang and Xu, 2025). This aligns with self-regulated learning theory, which posits that students with higher perceived competence are better able to monitor their learning and regulate their use of AI, thereby avoiding over-reliance (Zimmerman, 2002). Importantly, the data suggest that in this context, a well-developed sense of competence primarily is positively associated with individuals’ critical and creative engagement with learning tools, while concurrently being negatively associated with the tendency toward overreliance (Capron Puozzo and Audrin, 2021; Kim and Kim, 2024). Interventions aimed at enhancing students’ technological self-efficacy, such as tiered training in prompt engineering, are therefore critical for fostering responsible usage behaviors (Kong et al., 2024).

### The direct effect of perceived relatedness on behavioral patterns (H3a and H3b)

The analysis confirms significant direct effects of perceived relatedness on the two behavioral patterns. It exhibits a significant positive impact on deep processing (*β* = 0.223) and a significant negative effect on excessive reliance (*β* = −0.144), verifying both direct effect hypotheses (H3b and H3a). This indicates that college students embedded in supportive social environments are more inclined to engage in deep thinking when using AI. Supportive social interactions, such as high-quality teacher-student and peer exchanges, foster a sense of social presence and positive interdependence, which are positively associated with deep cognitive engagement and collaborative knowledge construction (Miao et al., 2022; Gheorghe et al., 2023). Furthermore, being part of a network of strong social relationships and a participatory classroom climate not only enhances social belonging but also provides the normative scaffolding and resources necessary for sustained, effortful processing of complex information (Schürer et al., 2025; Zheng et al., 2025). Concurrently, the social pressure inherent in these relationships can motivate students to more carefully verify GAI outputs (Hohenstein et al., 2023). Perceived relatedness fosters deeper cognitive engagement by providing students with a sense of security that encourages more effortful information processing (Furrer and Skinner, 2003). According to socio-technical theory (Bostrom and Heinen, 1977), such strong social relationships and collaborative norms can effectively restrain the one-way dominance of the technical system, thereby providing a direct social mechanism that alleviates excessive reliance. This underscores the importance of building collaborative learning communities, for instance through peer review and group discussions, to facilitate collective validation of GAI outputs and thereby deepen cognitive engagement (Inker et al., 2023).

### The mediated path of perceived autonomy through intrinsic motivation (H4a and H4b)

Path analysis indicates that perceived autonomy significantly and positively predicts intrinsic motivation (*β* = 0.211), which subsequently serves as a significant driver of both behavioral outcomes. This enhanced intrinsic motivation, in turn, channels behavior by reducing the tendency toward excessive reliance and promoting engagement in deep processing. Further analysis of the indirect effects revealed that perceived autonomy, by enhancing intrinsic motivation, is negatively associated with excessive reliance (*β* = −0.038, *p <* 0.05) and a significant positive influence on deep processing (*β* = 0.053, *p <* 0.05). This pattern of results supports the proposed mediated pathways (H4a and H4b). When college student users perceive control over the GAI interaction process, this satisfaction of autonomy needs activates the reward circuitry associated with exploratory behavior, thereby enhancing their intrinsic drive to engage with the technology (Ryan and Deci, 2000a, 2000b; Liu et al., 2022). This is consistent with recent findings that satisfying the need for autonomy can “plant the seed” of epistemic curiosity, a potent form of intrinsic motivation that drives individuals to seek and integrate new knowledge (Puerta-Sierra and Puente-Díaz, 2024). This internal drive, in turn, functions as a key self-regulatory mechanism. It reduces the tendency to seek quick, unreflective answers (An et al., 2024) by fostering a mastery-oriented mindset linked to critical thinking and deeper cognitive processing strategies (Ossa et al., 2023; Kwarikunda et al., 2022). This mindset may be reinforced by students’ need for cognition, a stable tendency toward effortful thinking that predisposes them to deeper processing (Cacioppo and Petty, 1982). Consequently, pedagogical environments that support autonomy and cultivate intrinsic motivation are consistently found to enhance critical engagement and sustainable learning outcomes, steering learners away from superficial dependence (Sari et al., 2021; Wang et al., 2024). The findings corroborate recent empirical work in technology-enhanced learning, confirming intrinsic motivation as a pivotal predictor of both the adoption of deeper processing strategies and superior learning outcomes (Poupard et al., 2025; Kwarikunda et al., 2022; Song and Zheng, 2024). Consequently, pedagogical strategies that cultivate intrinsic motivation—such as providing meaningful choices and avoiding excessive control—not only enhance learning interest but also indirectly guide students toward responsible GAI usage (Hulleman and Harackiewicz, 2009).

### The mediated path of sense of competence through intrinsic motivation (H5a and H5b)

The results support the mediated pathway involving sense of competence. It significantly promotes intrinsic motivation (*β* = 0.375), which in turn serves as a potent mediator, leading to reduced excessive reliance and increased deep processing. The analysis of indirect pathways further confirmed that a sense of competence, through its positive effect on intrinsic motivation, led to a significant reduction in excessive reliance (*β* = −0.068, *p <* 0.01) and a significant promotion of deep processing (*β* = 0.094, *p <* 0.01). These findings substantiate the proposed indirect effects (H5a and H5b). The strong positive impact of competence on intrinsic motivation may be attributed to the users’ perception of their own abilities affecting their level of trust and exploratory drive towards AI (Bansal et al., 2021). Specifically, a robust sense of self-efficacy can foster trust in technology and motivate more exploratory use, as observed in contexts from mobile payments to AI-assisted learning (Tian and Chan, 2024; Kim and Kim, 2024). This enhanced motivation, in turn, channels cognitive and behavioral engagement. Within educational technology settings, learners with higher intrinsic motivation, which often stems from greater self-efficacy, demonstrate greater attentional focus on critical information and achieve better learning performance through generative AI tools (Wang et al., 2022; Shahzad et al., 2025; Tokan and Imakulata, 2019). This positive link between internal motivation and meaningful outcomes is robust across domains. For instance, research in the context of work has similarly shown that internal regulation strongly predicts the experience of meaningful work (Allan et al., 2016). The internal drive cultivated by a sense of competence fosters a mindset oriented toward mastery and understanding, which is closely linked to critical thinking and deeper cognitive strategies (Ossa et al., 2023; Kwarikunda et al., 2022; Wang and Chang, 2022), thereby steering engagement away from superficial dependence. This suggests that educators can enhance students’ sense of competence by designing progressively challenging tasks and providing opportunities for successful experiences, thereby fueling intrinsic motivation and promoting deep engagement with GAI (Zapata-Cáceres and Martín-Barroso, 2021).

### The mediated path of perceived relatedness through intrinsic motivation (H6a and H6b)

The path analysis indicates a significant mediated pathway for perceived relatedness. It demonstrates a significant positive impact on intrinsic motivation (*β* = 0.227), which subsequently operates as a critical mechanism, translating into a negative effect on excessive reliance and a positive effect on deep processing. Examination of the indirect effects indicated that perceived relatedness, by bolstering intrinsic motivation, is negatively associated with excessive reliance (*β* = −0.041, *p <* 0.05) and a significant positive impact on deep processing (*β* = 0.057, *p <* 0.05). This set of findings supports the hypothesized indirect effects (H6a and H6b). College students who maintain harmonious social relationships within the GAI usage context experience enhanced intrinsic motivation, as the Unified Theory of Acceptance and Use of Technology (UTAUT) identifies social influence as a key factor affecting technology adoption willingness (Venkatesh et al., 2003). In technology-mediated learning environments specifically, fostering a sense of social relatedness through structured peer interaction directly enhances students’ intrinsic motivation (Lu et al., 2022), aligning with the broader understanding of social norms as motivators in digital contexts (Kim and Yoon, 2021). This motivation, enriched by social connectedness, then guides behavioral outcomes. Intrinsic motivation orients students toward learning goals rather than performance goals, making them more likely to engage deeply and resist superficial shortcuts (Vansteenkiste et al., 2004). It reduces the tendency for quick, unreflective reliance on AI by fostering a more deliberate and mastery-oriented approach (An et al., 2024). Simultaneously, it translates into more reflective and effortful cognitive engagement with GAI outputs, as intrinsically motivated learners are more likely to employ deep processing strategies (Sins et al., 2008). The perceived support from the social environment and the quality of interpersonal interactions are thus crucial, as they help shape the intrinsic motivation that drives deeper engagement and mitigates overdependence (Li and Liu, 2025; Rezaev and Tregubova, 2025). Hence, fostering supportive teacher-student and peer networks can, by bolstering intrinsic motivation, effectively steer students away from over-reliance and toward critical engagement with GAI (Prasad and Sane, 2024).

### Synthesis and model presentation

**Figure 2:**
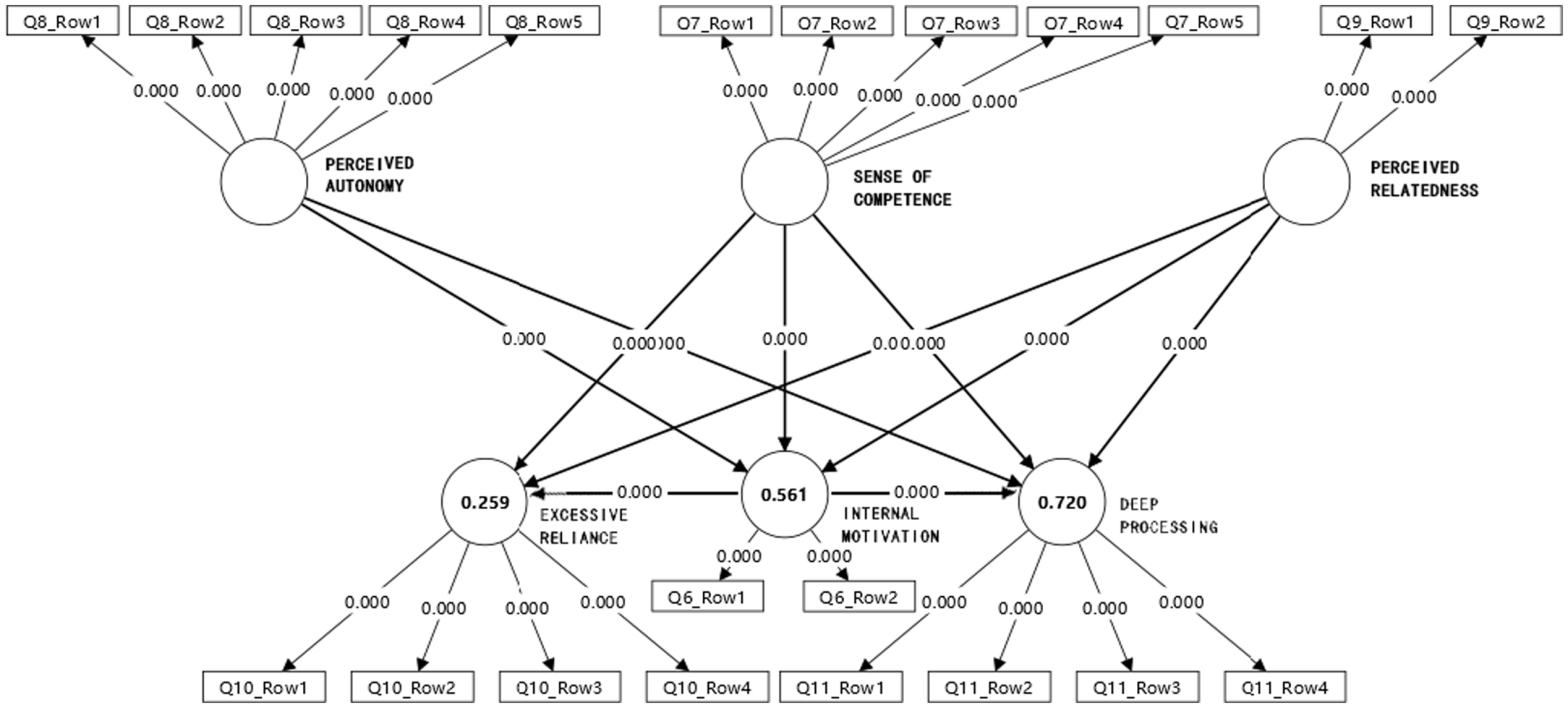
Revised model of influencing factors on college students’ GAI usage behavior. This figure presents the revised structural equation model, showing significant predictive paths from perceived autonomy, competence, and relatedness to the two behavioral outcomes: excessive reliance and deep processing in GAI usage.

To more intuitively present the results of the hypothesis testing, this study constructed a revised model of the influencing factors of college students’ GAI usage behavior patterns, as shown in Figure 2.

### Limitation

Several limitations of this study should be acknowledged, relating to the self-report nature of the data, the cross-sectional design, the cultural context, and the sampling characteristics.

First, the reliance on self-report data may introduce biases affecting the findings. Respondents might have overestimated or underestimated their GAI usage due to social desirability or inaccurate self-perception. Self-report measures are inherently susceptible to response biases, despite the validity tests conducted suggesting acceptable measurement properties. Such biases are particularly relevant when measuring socially sensitive behaviors like over-reliance, which students may be reluctant to acknowledge. Future research could address this through mixed-method designs combining surveys with behavioral data, including logging GAI interaction frequency, prompt complexity, and content modification patterns. These objective measures would provide more objective measures of deep processing versus over-reliance. Additionally, experience sampling methods could capture real-time usage patterns, reducing recall bias. Experimental designs and longitudinal tracking of usage logs could further complement self-report data, offering a more comprehensive understanding of students’ actual GAI usage behaviors across different learning contexts and tasks.

Second, limitations of the cross-sectional study. While our model identifies significant associations between psychological needs, intrinsic motivation, and GAI usage behaviors, these relationships should be interpreted as correlational, and it remains possible that reverse causation or third variables explain some findings. Although structural equation modeling can infer structural relationships based on theoretical assumptions, cross-sectional data cannot dynamically capture the evolutionary process of variables over time. For instance, students who engage in deep processing may subsequently develop stronger perceptions of competence. Future research may employ longitudinal designs to examine the dynamic interplay between need satisfaction and behavioral patterns across different stages of learning. Tracking students across an academic term or even across multiple semesters could reveal how changes in perceived autonomy, competence, and relatedness correspond to shifts in GAI usage patterns. Experimental studies are also warranted, where psychological needs are manipulated in controlled settings to establish causal effects on deep processing and over-reliance. Such designs would provide stronger evidence for the directional relationships hypothesized in our model.

Third, the contextual specificity may limit generalizability. The sample was predominantly composed of Chinese university students, representing a specific cultural and educational background. Perceptions of autonomy, competence, and relatedness, and their relationships with technology use, may be understood and expressed differently across cultural contexts. For example, students from different cultural backgrounds might experience these psychological needs in ways that reflect their particular social norms and values, which could potentially lead to variations in GAI engagement patterns. In some cultural settings, the role of relatedness in shaping technology use might be more pronounced than observed here, while the expression of autonomy could take different forms. Future research could usefully examine this model in diverse international settings to explore its applicability across cultural contexts. Cross-cultural comparative studies would help clarify which aspects of the model hold consistently across cultures and which may be more context-dependent, contributing to a more nuanced understanding of how cultural factors relate to the motivational mechanisms underlying GAI usage.

Fourth, the sampling characteristics present limitations regarding representativeness. Although the sample size was adequate and efforts were made to achieve diversity, several sampling constraints should be noted. First, the sample was drawn primarily from Chinese universities, and while regional diversity was achieved within China, the absence of cross-national comparisons limits our understanding of how different educational systems, policy environments, and cultural values shape GAI adoption patterns. Second, the sample included relatively fewer graduate and doctoral participants. While this distribution reflects the target population, it may limit the applicability of findings to advanced degree seekers who may have different GAI usage patterns due to their research-intensive academic demands, which often involve more complex information synthesis and original knowledge production. Third, the disciplinary distribution showed some concentration in certain fields, with education and humanities disciplines being relatively overrepresented compared to others fields. Although the sample covered a range of disciplinary categories, the uneven representation across fields may affect the generalizability of findings to students in different academic domains. Future research should strive for more balanced sampling across academic levels and disciplinary backgrounds, and include international comparisons to enhance the robustness and generalizability of the findings. Stratified sampling strategies could be employed to ensure proportional representation across key demographic and academic dimensions, enabling more fine-grained analyses of group differences in GAI usage behaviors.

### Summary and suggestions

Based on self-determination theory, this study developed a model of factors influencing college students’ GAI usage behavior and empirically tested it with 750 participants, yielding the following key findings. The findings reveal that students exhibit two distinct behavioral patterns in their GAI usage: over-reliance behaviors, such as directly copying GAI-generated answers without modification, turning to GAI for help whenever encountering problems, and adopting GAI outputs uncritically; and deep processing behaviors, characterized by designing prompts to elicit optimal responses, carefully evaluating the accuracy of GAI-generated content, selectively using information, and revising or refining original answers based on GAI suggestions. These behavioral patterns are shaped by both direct and indirect pathways through intrinsic motivation. Core psychological needs are key predictors of behavioral patterns. Specifically, perceived competence is positively associated with intrinsic motivation, effectively curbing excessive reliance while fostering deep processing. This indicates that confidence in one’s abilities is crucial for avoiding technological dependency and achieving critical engagement. Perceived autonomy substantially promotes deep processing, suggesting that granting students autonomy in technology choices can effectively stimulate reflective usage. Perceived relatedness regulates both behavioral types, underscoring the guiding role of interpersonal interaction in technology’s rational application. Notably, intrinsic motivation demonstrates a significant direct effect, promoting deep processing while curbing excessive reliance. This suggests its role as a key driver of self-determined GAI usage alongside the basic psychological needs. This reveals a transformation barrier between enhanced motivation and behavioral change, necessitating direct intervention through strengthening core psychological needs to modify behavioral patterns.

Based on self-determination theory, this study systematically validates the applicability of the theory to GAI learning behaviors while refining the explanatory boundaries of traditional motivation theories. Practically, these findings offer concrete implications for multiple stakeholders in higher education, informing how educators design learning tasks that foster autonomy and deeper engagement, how administrators develop policies and training programs that promote responsible GAI use, and how technology designers create tools that enhance students’ sense of control, confidence, and social connection. It provides a foundation for designing targeted interventions, emphasizing prioritized technical competence development supplemented by autonomy empowerment and collaborative ecosystem building, with the aim of normalizing deep processing behaviors. Drawing on empirical findings regarding influencing factors and mechanisms, systematic intervention strategies are proposed across four interconnected dimensions: educational practice, technical design, policy management, and collaborative cultivation.

### Educational practice: constructing a need-supportive learning ecosystem

Based on empirical findings, this study identifies perceived competence as pivotal for curbing excessive reliance and fostering deep processing. To optimize interventions, this study also highlights the importance of addressing potential risk patterns. These findings necessitate constructing a stratified educational intervention ecosystem. First, establish a hierarchical curriculum system. Lower-grade students should receive foundational training in prompt design principles, GAI output cross-validation techniques, and ethical risk identification to build technological control. Senior students require advanced courses covering GAI-assisted research design, critical integration frameworks for multi-source information, and strategies to address GAI illusions to bridge ability gaps through academic problem-solving. Second, cultivate dynamic error-correction awareness. Leveraging evidence that error-correction practices deepen cognition (Cai et al., 2016), educators should guide students to maintain personal “GAI error case databases” documenting issues like factual deviations. Students must then complete diagnostic correction reports, such as analyzing ChatGPT’s false citations, proposing verification methods, and transforming errors into competence-building opportunities. Third, implement interventions for motivational decline. Applying utility value intervention theory (Hulleman and Harackiewicz, 2009), educators should deploy contextualized tasks like AI-centric academic activities to reactivate motivation. Concurrently, establish academic integrity benchmarks incorporating “AI transparency statements”, requiring annotation of prompt iterations, content revision logic, and clarification of GAI limitations versus personal contributions, compelling deeper cognitive engagement. Finally, consider designing collaborative tasks that align with the construct of perceived relatedness (e.g., interdisciplinary prompt optimization or cross-peer review of GAI reports). Such tasks can foster the social processing that supports deep learning, while incorporating systematic questioning training to help all students, particularly those prone to default trust, critically evaluate AI outputs and mitigate over-reliance.

### Technical design: building a “guided” GAI tool

Given that intrinsic motivation alone may be insufficient to fully counteract the risk of excessive reliance in all contexts, overcoming this bottleneck requires developing intelligent tools integrating “autonomy-competence-relatedness” needs. First, implement a competence growth feedback system. A dynamic skill panel should visualize core ability metrics (e.g., prompt design quality, risk identification accuracy, cross-source verification frequency). Capitalizing on competence’s predictive power for deep processing, the system can push step-by-step challenges to high-risk users, offering prompts such as: “Prompt score: B. Attempt designing clinical diagnosis prompts requiring multi-step reasoning.” Drawing from self-regulated learning frameworks (Prasad and Sane, 2024), a rhetorical interaction mechanism should trigger reflection prompts when unmodified text pasting is detected (e.g., “What theoretical foundations support this conclusion? Are there opposing viewpoints?”), forcibly blocking excessive dependence pathways. Second, design an autonomy-enhancing control module. Introduce output modes with adjustable support intensity: lower-grade students access framework-assisted modes to address knowledge gaps, while seniors utilize heuristic modes to counter motivation decline. Provide weekly dependence analytics reports coupled with “deep processing strategy packages” (adversarial prompt templates, cross-validation flowcharts), aligning with Bayesian learning principles for autonomy support (Dettweiler et al., 2017). Enhance risk warnings for sensitive content (e.g., “23% statistical bias risk—verify Nature’s 2024 review”) to compensate for identification gaps. Third, build a relational social verification network. Create a one-click help system with “peer/faculty review” channels for rapid query resolution, activating relatedness’s role in deep processing. Enable collaborative real-time editing of AI outputs to strengthen social processing.

### Policies and management: establishing a hierarchical support and constraint framework

Grounded in self-determination theory, this study clarifies the key mechanisms that shape college students’ GAI learning behaviors. To translate these theoretical insights into practical effectiveness and address the nuanced needs of diverse learners, it is imperative to establish a hierarchical support and constraint framework that coordinates university-level planning with teacher-led implementation and establish a hierarchical support and constraint framework that coordinates university-level overall planning and teacher implementation at the policy and management level, precisely intervening in the key pain points of different groups. First, at the university level, it is necessary to strengthen standards, resources, and norms. Universities should clarify GAI literacy standards (Su et al., 2025), anchor ability development goals, and explicitly incorporate the core elements of deep processing ability into college students’ graduation requirements or core competency indicator systems. This provides a clear benchmark for ability development for all teachers and students, guiding teaching practice to focus on ability cultivation rather than tool use itself. Meanwhile, universities should implement differentiated resource allocation, precisely empowering weak links, and tilting policy resources toward key groups. For example, targeted “Workshops on In-depth Application of GAI and Critical Thinking in Academic Scenarios” can be offered; “Mentorship-based GAI Counseling” or “Cognitive Deepening Training Camps” can be provided to groups at higher risk of reliance, ensuring that support measures accurately reach the groups with the most urgent needs. In addition, universities should update academic integrity norms, clearly defining the boundaries between reasonable use of GAI, excessive reliance, and academic misconduct, and particularly emphasizing the two core requirements of “transparency” and “substantive contribution” in academic norms. For instance, students can be required to clearly indicate the use of GAI when submitting results, and elaborately explain the critical review, revision, integration, and original contributions they have made to the content generated by GAI. Second, at the teacher level, the focus should be on design, evaluation, and demonstration. Schools should provide professional development support for teachers, training them to design learning tasks that can effectively enforce or encourage deep processing behaviors. For example, applying the requirement of process explicitness in the 6P teaching method (Plan-Prompt-Preview) (Kong et al., 2024), students can be required to submit records of prompt iteration and optimization, comparative analysis reports between GAI’s original output and their final drafts, and explanations of the verification process for key points in GAI-generated content in their assignments. Teachers should significantly increase the evaluation weight of the “process” of GAI use in the course grading criteria, rather than merely focusing on the final results. According to the quantitative evaluation standards for the process of GAI use in the academic writing assessment framework (McNutt et al., 2018), emphasis should be placed on evaluating the quality of students’ prompt design, the rigor of methods for verifying GAI output, and the depth and creativity in integrating GAI-generated content into their own knowledge systems or solutions. In addition, teachers should pay attention to strengthening positive demonstration and feedback, actively demonstrating and elaborating on how they use GAI in depth and critically to complete teaching or research work in courses, providing students with real and high-level demonstration cases. At the same time, teachers are required to give timely, specific, and public positive feedback on students’ deep processing behaviors in tasks, such as high-quality prompt design, rigorous verification behaviors, and effective integration and re-creation. This demonstration and feedback mechanism helps create a positive classroom culture of “critical use of GAI”, directly strengthens the deep processing behaviors emphasized in the study, and leverages the positive role of “perceived relatedness.”

### Cultural construction: fostering a shift from instrumental to value rationality

This study reveals that college students’ over-reliance on GAI fundamentally stems from a value deviation, rendering their usage instrumental and superficial. Path analysis shows perceived relatedness significantly influences behavioral patterns through intrinsic motivation, with relatedness reflecting collective perceptions of value (Chirkov et al., 2003; Wu and Yang, 2025). Thus, skill training and institutional constraints alone cannot address over-reliance. A cultural shift from instrumental to value rationality is urgently needed. First, advocate “critical collaboration” to redefine human-AI relationships. Universities should convey that “GAI is a thinking partner, not an answer machine” through orientation and academic integrity courses. Students must recognize deep processing as cognitive dominance in human-AI collaboration. When students understand that questioning, verifying, and reflecting constitute true competence, intrinsic motivation is stimulated, leading them toward deep processing over shortcuts. Second, construct a value-oriented evaluation culture incorporating process engagement into recognition. The prevalent “outcome worship” breeds over-reliance (Zhang et al., 2024). This study confirms intrinsic motivation as the key mediator between perceived needs and behavior, requiring value recognition support. Awards, scholarships, and graduate recommendations should recognize process engagement, guiding students from “pursuing correct answers” to “pursuing cognitive growth.” Third, cultivate rational public opinion to resist technological mythmaking. Media glorification of GAI’s “omnipotence” exacerbates instrumental dependence (Ballatore and Natale, 2023). Universities should proactively analyze GAI’s capabilities and risks through campus media and dialogues, fostering prudent attitudes. When “rational GAI use” dominates campus discourse, students’ perceived relatedness is fulfilled through cultural identification, aligning behavior with collective expectations.

## Data Availability

The raw data supporting the conclusions of this article will be made available by the authors, without undue reservation.
